# Developing a novel therapeutic and bioactive resin-based root canal sealer incorporated with silver polydopamine-modified hydroxyapatite fillers

**DOI:** 10.1186/s12903-025-06469-2

**Published:** 2025-07-09

**Authors:** Samar M. Nashaat, Enas T. Enan, Ahmed S.G. Srag El-Din, Dina Abdelaziz

**Affiliations:** 1 Department of Dental Biomaterials, Faculty of Dentistry, Mansoura University, 35516 Mansoura, Egypt; 2 Department of Pharmaceutics, College of Pharmacy, Almaaqal University, Basrah, 61014 Iraq; 3 Department of Pharmaceutics, Faculty of Pharmacy, Delta University for Science and Technology, Gamasa, 11152 Egypt

**Keywords:** Antibacterial, Hydroxyapatite, Polydopamine, Remineralization, Root canal sealer, Silver nanoparticles, Wettability

## Abstract

**Background:**

Residual root canal microbiota in the inaccessible areas of the root canal despite cleaning and shaping and the adverse effects of the various root canal irrigants on dentin mineral content pose a major challenge to successful root canal therapy. The aim of this study was to develop therapeutic and bioactive resin-based root canal sealer (RCS) with good wetting ability.

**Methods:**

Silver polydopamine-modified hydroxyapatite (HA-PDA-Ag-PDA) fillers were synthesized. Hydroxyapatite particles were analyzed using X-ray diffraction (XRD), while the final prepared fillers were characterized by Fourier transform infrared (FT-IR) spectroscopy and transmission electron microscopy (TEM). The resin-based RCS (Adseal^®^) served as the control group (G0). Two concentrations of HA-PDA-Ag-PDA fillers (1 wt% and 2 wt%) were incorporated into the tested RCS (G1 and G2), respectively. The antibacterial properties, mineralization, and wettability of unmodified RCS (G0) and modified RCS (G1 and G2) were evaluated. The data were analyzed by one-way ANOVA, followed by a post hoc test for pairwise comparisons.

**Results:**

XRD, FT-IR, TEM characterizations confirmed the successful synthesis of the HA-PDA-Ag-PDA fillers. Modified RCS showed strong bacterial inhibition effect against E. faecalis with insignificant difference between G1 and G2. Modified RCS (G1 and G2) also showed good mineralization potential and improvement in wettability of dentin surface.

**Conclusions:**

HA-PDA-Ag-PDA fillers significantly improved the antibacterial, mineralization properties as well as the wetting ability of resin-based RCS.

## Background

Appropriate selection of endodontic sealer is an essential factor for the success of root canal therapy [1]. Endodontic sealers (RCSs) are classified based on their main chemical composition: zinc oxide eugenol, glass ionomer, calcium hydroxide, resin-based, and bioceramic-based sealers [2]. No sealer satisfies all the criteria of an ideal root canal sealer to ensure successful root canal treatment. Epoxy resin-based sealers (ERBSs) are considered the gold standard due to their superior physicochemical features and biological responsiveness. They generally exhibit good dimensional stability, radiopacity, adequate film thickness, low solubility, good flow characteristics and high sealing capacity. In addition to having low toxicity, they can adhere to dentin [3]. Nevertheless, their primary drawbacks include limited antimicrobial properties and inability to biomineralize and exhibit bioactivity. Furthermore, the hydrophobic nature of ERBSs may affect their wetting ability to dentin surface, affecting their penetration into the fine areas of the root canal system, which in turn affects the sealing ability and antibacterial effectiveness of RCS [4]. 

One of the challenges of root canal therapy is that, it is impossible to completely fully eliminate microorganisms from the root canal and accessory canals, resulting in refractory infections and chronic apical periodontitis following endodontic therapy [5]. *Enterococcus faecalis (E. faecalis*), a gram-positive facultative anaerobe, is strongly associated with persistent periapical infections, with its presence detected in 23–77% cases of unsuccessful endodontic therapy [6]. Enhancing endodontic sealers with antimicrobial nanoparticles is one way to neutralize any germs and improve their antibacterial effects, enhancing endodontic success and prognosis. Silver nanoparticles (AgNPs) are one of the most widely utilized nanomaterials in the biomedical and dentistry due to their antimicrobial properties. Their distinctive capability to create various nanostructures, and their reasonably low production cost, make them extremely important in the field of nanomedicine [7]. 

Another challenge during the endodontic treatment is the adverse effects of endodontic irrigants such as, sodium hypochlorite (NaOCl), ethylenediamine tetra acetic acid (EDTA), calcium hydroxide paste, and chlorhexidine gluconate solution (CHX) on dentin mineral content [8, 9]. It was shown that these solutions can damage the radicular dentin structurally and mechanically making it more brittle and prone to fracture [10]. In order to counteract the effects of demineralizing irrigation solutions, improve dentin hardness, addition of remineralizing nanoparticles to root canal filling systems could be the solution [11, 12]. The high quantities of remineralizing ions present close to the dentin surface may encourage remineralization, which strengthens the endodontically treated tooth and establish a more stable tooth-material contact, increasing the sealing ability of these cements [13]. Hydroxyapatite is often used as a remineralizing agent for enamel and dentin because of its calcium and phosphate composition, which is similar to that of human hard tissues [14]. When compared to standard HA, nano-hydroxyapatite (nano-HA) has gained a lot of interest because of its tiny size, it has a greater reaction surface and exhibits better bioactivity when compared to bigger crystals [15]. 

Inspired by the long-lasting adhesion phenomenon of mussels to any surface in a humid environment using a protein released by the mussel byssus, the wet-adhesion is now widely used in dental research as a surface tissue adhesive and biomaterial surface modification. Polydopamine (PDA) is the synthetic counterpart replicating the unique adhesive properties of the mussel-adhesive-foot-proteins (Mefps) [16]. It has lately been recognized as one of the most powerful tools for surface modification due to its ease of use, versatility, and biocompatibility [17]. Combining nano-HA with PDA and Ag presents a simple biologically inspired approach for the modification of nanohydroxyapatite.

Consequently, the current study presents a commercial resin-based root canal sealer incorporated with synthetic silver polydopamine-modified hydroxyapatite (HA-PDA-Ag-PDA) fillers, welling to impart antibacterial and bioactive properties and to improve the wettability of the sealer to dentin surface. The study’s null hypothesis is that the addition of HA-PDA-Ag-PDA filler has no influence on the antibacterial, mineralization nor wettability properties of the resin-based root canal sealer.

## Materials and methods

Following approval number (M0109023DM) of ethical committee of Faculty of dentistry, Mansoura University, fifteen single rooted teeth were gathered for root canal sealer wettability evaluation. The study was performed using one commercial RCS, Adseal^®^ (Meta Biomed, Cheongju, South Korea).

### Sample size calculation

Based on G Power software version 3.1.9.7 with an effect size of 0.864, employing a 2-tailed test, with an α error of 0.05 and a power of 80%, the sample size was established with at least 6 per group. The sample size was increased to 10 per group.

### Synthesis of nano-hydroxyapatite powder (nano-HA)

The wet chemical precipitation method was utilized to prepare hydroxyapatite particles. 100 ml of distilled water was used to dissolve 2.35 g of calcium nitrate tetrahydrate and the pH was modified to about 9–10 by using ammonium hydroxide solution. Afterwards, another 100 ml was used to dissolve 0.771 g of ammonium hydrogen phosphate, and then added dropwise to Calcium nitrate solution while being stirred for 24 h at ambient temperature forming a gelatinous precipitate. The resulting solution was aged for another 24 h and the formed HA powder separated by centrifugation (TM1721, Fisher Scientific Company, U.S.A). Distilled water was used to wash the resultant powder multiple times and dried an oven at 50^o^C overnight [18, 19]. 

### Synthesis of silver polydopamine-modified HA fillers (HA-PDA-Ag-PDA)

The nano-HA particles were distributed in 100 mL of Tris solution, and the the pH was modified to about 8.5 using Hydrochloric acid. Dopamine hydrochloride was subsequently included to produce an aqueous solution with a 2 mg/mL concentration, while stirring it for 24 h at ambient temperature. The solution was left open so that dopamine hydrochloride could oxidatively self-polymerize to form a PDA coating on the surface of nano-HA. The resultant powder was undergoing centrifugation and rinsed with ethanol and deionized water three times, then dried out for 24 h at ambient temperature. HA-PDA was distributed in 100 mL of silver nitrate solution (50 mM) and stirred for 4 h at ambient temperature to produce HA-PDA-Ag. The catechol and amino groups on the PDA coating would facilitate Ag^+^ adsorption onto HA-PDA, which would subsequently undergo in situ conversion to metallic silver nanoparticles. The product was centrifuged, rinsed with ethanol and deionized water three times, then dried. For second PDA coat, HA-PDA-Ag was redispersed in an aqueous dopamine hydrochloride solution and stirred continuously for 4 h. The final product was separated then dried with the same method as previously mentioned [20]. 

### Characterization of prepared HA-PDA-Ag-PDA fillers

Confirmation of the preparation of (nano-HA) was achieved by determining its functional groups by Fourier Transform Infrared Spectroscopy (Jasco FT/IR 4100) while the crystalline phase was investigated using an X-ray powder diffractometer (XRD, BRUKER Co.D8Advanced, Germany). The functional groups of each HA-PDA, HA-PDA-Ag and HA-PDA-Ag-PDA were determined by FTIR, while the morphology and the size of the final synthesized filler was examined by transmission electron microscope (TEM) (Talos L120C G2, Thermo Fisher Scientific, USA).

### Specimens’ preparation and grouping

The self-cured ERBS Adseal^®^ (Meta Biomed, Cheongju, South Korea) served as a control group (G0). It was compared to the modified groups (G1) and (G2) where silver polydopamine-modified HA fillers were added to the resin-based sealer with two concentrations by weight 1% and 2%wt respectively. The antibacterial activity, remineralization potential, and wettability of each group were evaluated.

For preparation of specimens, HA-PDA-Ag-PDA fillers were incorporated into the Adseal RCS according to a standardized protocol predicated on the incorporation of weight% of particles. The essential weight of nanoparticles was measured for each gram of RCS using a four digits sensitive balance (RADWAG Wagi Elecktroniczne, model AS 220R1, Poland, EU) to attain the required concentration of nanoparticles relative to RCS. HA-PDA-Ag-PDA fillers were added to RCS and mixed with a plastic flat ended dental spatula until a creamy homogenous mix is obtained.

### Evaluation of antibacterial properties

#### Microbial strain and growth media

The antibacterial efficacy was evaluated against Enterococcus faecalis (ATCC 29212). Brain heart infusion (BHI) broth was inoculated with E. faecalis strains from the stock culture and incubated for 24 h at 37° C. Bacterial proliferation was evaluated by the observed turbidity in the broth after incubation. The inoculum tube was visually compared to the standards to evaluate turbidity. BHI agar was utilized as a culture medium for the modified direct contact test.

#### Modified direct contact test (MDCT)

The MDCT involves quantifying the colony-forming units (CFU) of bacterial proliferation in 96-well microtiter plates [21]. A uniform coating of the tested sealer was applied to the well floors (Ten wells for each group). A total number of 60 specimens were prepared, 30 fresh specimens were tested immediately, and 30 specimens were tested after 24 h (10 for each group). A 10 µL of a bacterial suspension of (10^5^ CFU/ml) cells was applied to the surface of the sealer specimen. Specimens were incubated for up to an hour at 37° C and 100% humidity until the suspension liquid evaporated, leaving a thin bacterial film in contact with the examined sealer’s surface. Each well containing the material received 240 µL of BHI broth. Following the bacterial suspension was gently mixed for one minute using a pipette, it was diluted and 10 µL portions were applied to BHI agar plates. The plates were then incubated at 37 °C for 24 h, after which the colonies on the plates were counted. The CFU/mL was calculated and compared [22]. 

### Evaluation of the mineralization potential

The mineralizing activity of the experimental RCS was evaluated by detecting the formation of hydroxyapatite on sealer surface using Scanning electron microscope fitted with energy dispersive X-ray (SEM/EDX) (Quanta FEG250, Thermo Fisher Scientific (FEI)) [23]. One representative disc shaped specimen for each group was made in a plastic split mold (10 mm in diameter and 2 mm in thickness) and left to set at ambient temperature set for 24 h, immersed in simulated body fluid (SBF), 10 ml for each specimen, at 37 °C which was refreshed every day. The surface morphology and the change in the levels of calcium and phosphorous ratios were examined by (SEM/EDX) at 14 and 28 days [24]. 

### Evaluation of the wettability

The wettability of the modified RCS to dentin was evaluated by measuring the contact angle between them using sessile drop technique. Fifteen single-rooted, sound human teeth were collected from the Oral Surgery Department clinic at the Faculty of Dentistry, Mansoura University. The collected teeth were previously extracted for periodontal reasons. They were cleaned using tap water and an ultrasonic scaler to remove any attached debris, then examined to exclude out teeth with root caries or defects. Then they were washed with 0.1% thymol solution for 24 h and stored in normal saline solution until used [25]. Thirty sections of dentin were obtained by longitudinally sectioning the teeth in a bucco-lingual direction with a microsaw, ten for each group. Each half was flattened and smoothed using silicon carbide paper with grit sizes 320, 600 and 1200. The longitudinal dentin slices were irrigated with NaOCl and EDTA solution replicating the chemomechanical preparatory irrigation. Using an endodontic spreader, a droplet with a diameter of approximately 1 mm was applied to the dentine specimen [26]. To determine the contact angle, an image was captured with a high-resolution camera immediately after deposition of the sealer’s drop onto dentin surface and analyzed using IC Measure software (IC Measure 2.0.0.245; The Imaging Source, Bremen, Germany) [27]. 

### Statistical analysis

The data’s normality was assessed using the Kolmogorov and Shapiro-Wilk tests, and its homogeneity was confirmed by Levene’s test. Statistical analysis was performed with SPSS 20^®^ (SPSS, IBM, USA). Group comparisons were performed with a One-Way ANOVA test, followed by Tukey’s Post Hoc test. A comparison between the fresh mix and the mixture after 24 h was performed with a paired t-test. The significant level was established at *P* < 0.05.

## Results

### Characterization of prepared HA-PDA-Ag-PDA fillers

#### X-ray diffraction analysis (XRD)

**Fig. 1:**
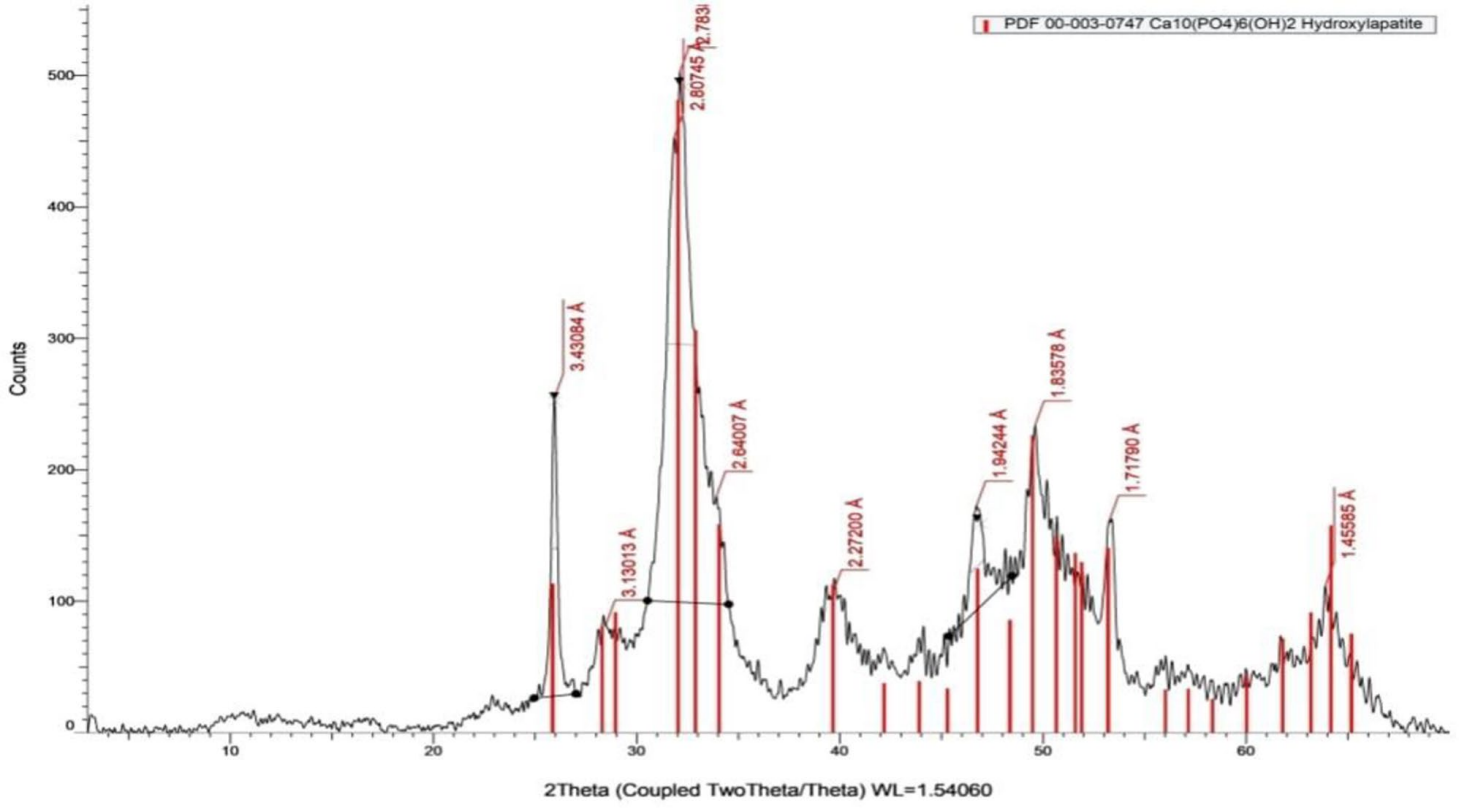
XRD pattern of HA

XRD patterns of synthetic hydroxyapatite powder, as shown in Fig. 1, have characteristic peaks comparable with the standard data reported by Joint Committee for Powder Diffraction (JCPDS, PDF # 09-0432, Hydroxyapatite, syn). Peaks occurred at 2Ɵ= 25.95°, 31.85°, 32.12°, and 49.61° which corresponds to the reflection peaks of crystalline HAp [28]. 

#### Fourier transformation infrared analysis (FTIR)

**Fig. 2:**
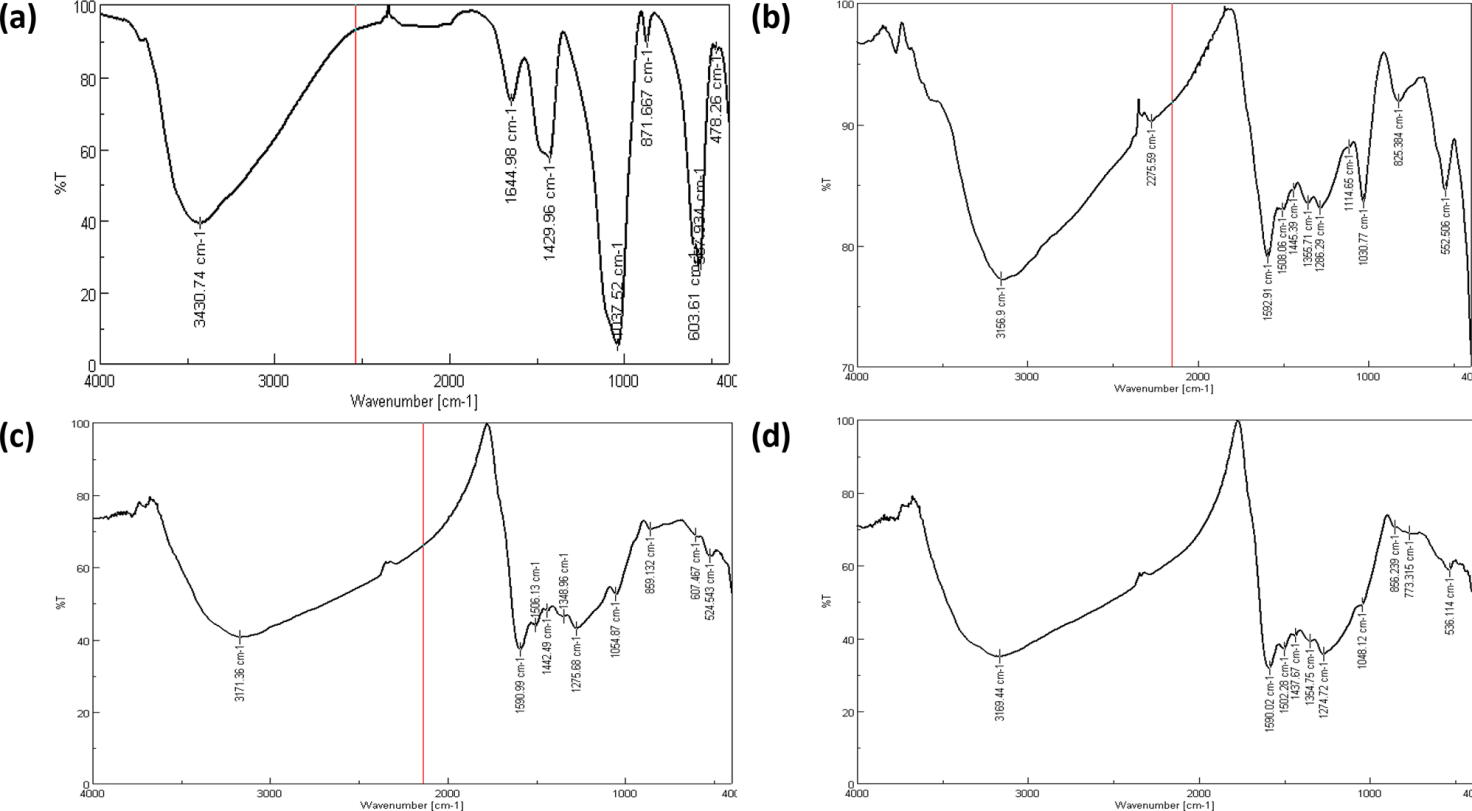
FTIR spectra of: (**a**) HA (**b**) HA-PDA (**c**) HA-PDA-Ag (**d**) HA-PDA-Ag-PDA

The FTIR spectrum has been collected between 400 and 4000 cm^− 1^. The FTIR spectra for each stage of the HA-PDA-Ag-PDA filler formation showed multiple distinct band peaks that correspond to various functional groups and interactions, as shown in Fig. 2. First, the FTIR spectrum of the pristine hydroxyapatite (HA) displayed characteristic peaks indicative of its composition. The prominent peak at 3430 cm^− 1^ indicated O-H stretching vibrations, which suggested the presence of hydroxyl groups on HA surface. Peaks at 1037 cm^− 1^, 603 cm^− 1^, 567 cm^− 1^ and 478 cm^− 1^ were consistent with phosphate (PO_4_^3−)^ vibrations. In addition, B type carbonate band also was seen around 1429 cm^− 1^, which indicated the presence of carbonate apatite. This may be due to absorption of carbon dioxide from the atmosphere [28]. Upon coating HA with polydopamine (PDA), notable changes were observed in the FTIR spectrum. While the O-H stretching peak remained, the broadening of this peak (3156.9 cm^− 1^) suggested possible hydrogen bonding or interaction between hydroxyl groups on HA and PDA. Peaks at 1592 cm^− 1^ and 1508 cm^− 1^ indicated C = C stretching vibrations and aromatic ring vibrations in PDA have been shown, demonstrating successful PDA coating [29]. 

The subsequent addition of silver nanoparticles (Ag) to the HA/PDA composite yielded a distinct FTIR spectrum. A prominent peak at 3171 cm^− 1^ indicated hydroxyl groups, possibly originating from both PDA and Ag. The persistence of peaks related to PDA (1590 cm^− 1^ and 1442 cm^− 1^) in HA-PDA-Ag implied that PDA may contribute to stabilizing or interacting with the silver nanoparticles. The slightly shifting of the peak at 1286 cm^− 1^, which were ascribed to the C–O bonds to lower wave numbers of 1275 cm^− 1^ could result from the creation of bonds between silver and oxygen. when PDA bonded to the surface of the silver nanoparticles [30]. 

Finally, a fourth layer of polydopamine was applied to the HA/PDA/Ag composite. The FTIR spectrum of HA-PDA-Ag-PDA revealed additional changes. A peak at 3169 cm^− 1^ confirmed the presence of hydroxyl groups, likely originating from the extra polydopamine layer. Peaks at 1502 cm^− 1^ and 1437 cm-1 indicated the continued presence of PDA. The appearance of a peak at 1354 cm^− 1^ suggested the involvement of organic materials.

#### Transmission electron microscope analysis (TEM)

**Fig. 3:**
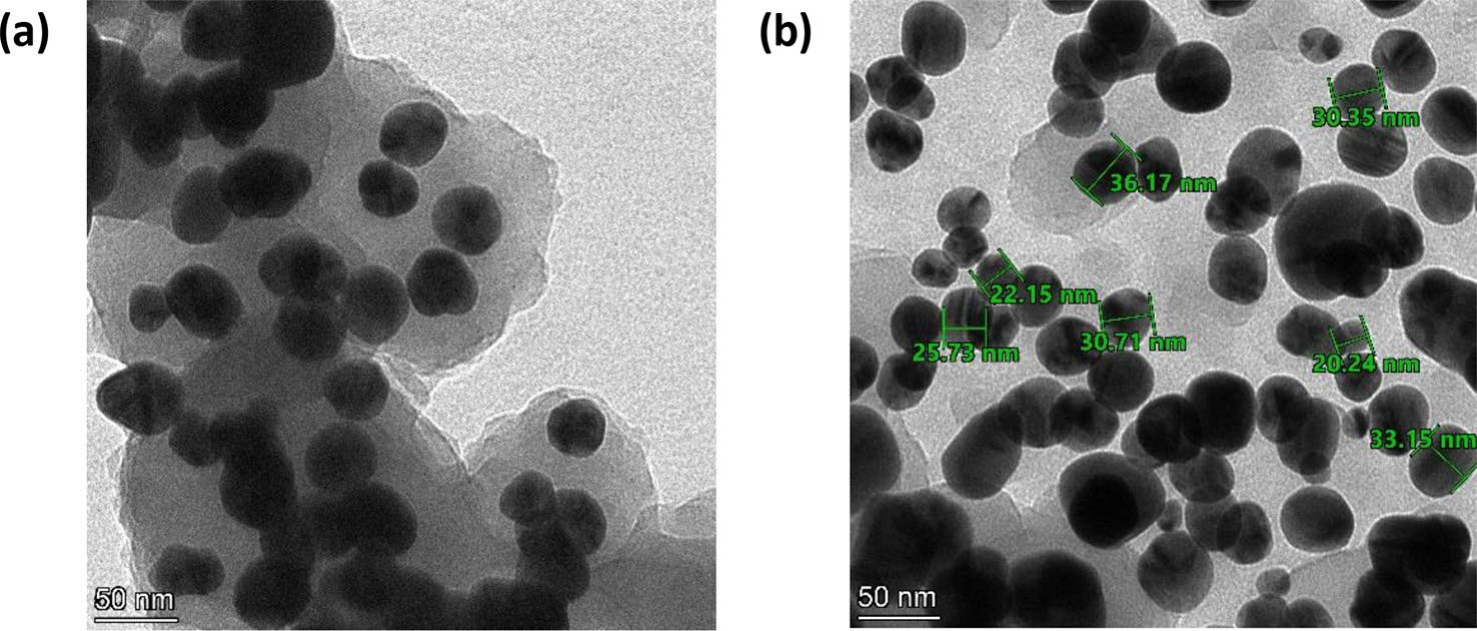
TEM images of: (**a**) HA-PDA-Ag-PDA (**b**) showing size of formed HA-PDA-Ag-PDA

TEM examination was used to investigate the shape and dimensions of filler particles. AgNPs were appeared as nanosized black spheres on the HA-PDA surface which ranges from 20 to 50 nm with a homogeneous dispersion and coated with a polymer-like material as shown in Fig. 3. The polymer coating was proven using FTIR analysis [20]. 

### Antibacterial properties

Regarding the antibacterial activity of unmodified RCS (G0) and modified RCS (G1 and G2) against E. faecalis, the means and standard deviations of the number of bacterial colonies for fresh mixed sealers and after 24 h are shown in Table 1. The MDCT indicated that the inclusion of HA-PDA-Ag-PDA fillers in RCS (G1 and G2) exhibited a statistically significant antibacterial activity against E. faecalis in both fresh mix and after 24 h (*p* < 0.05).

**Fig. 4:**
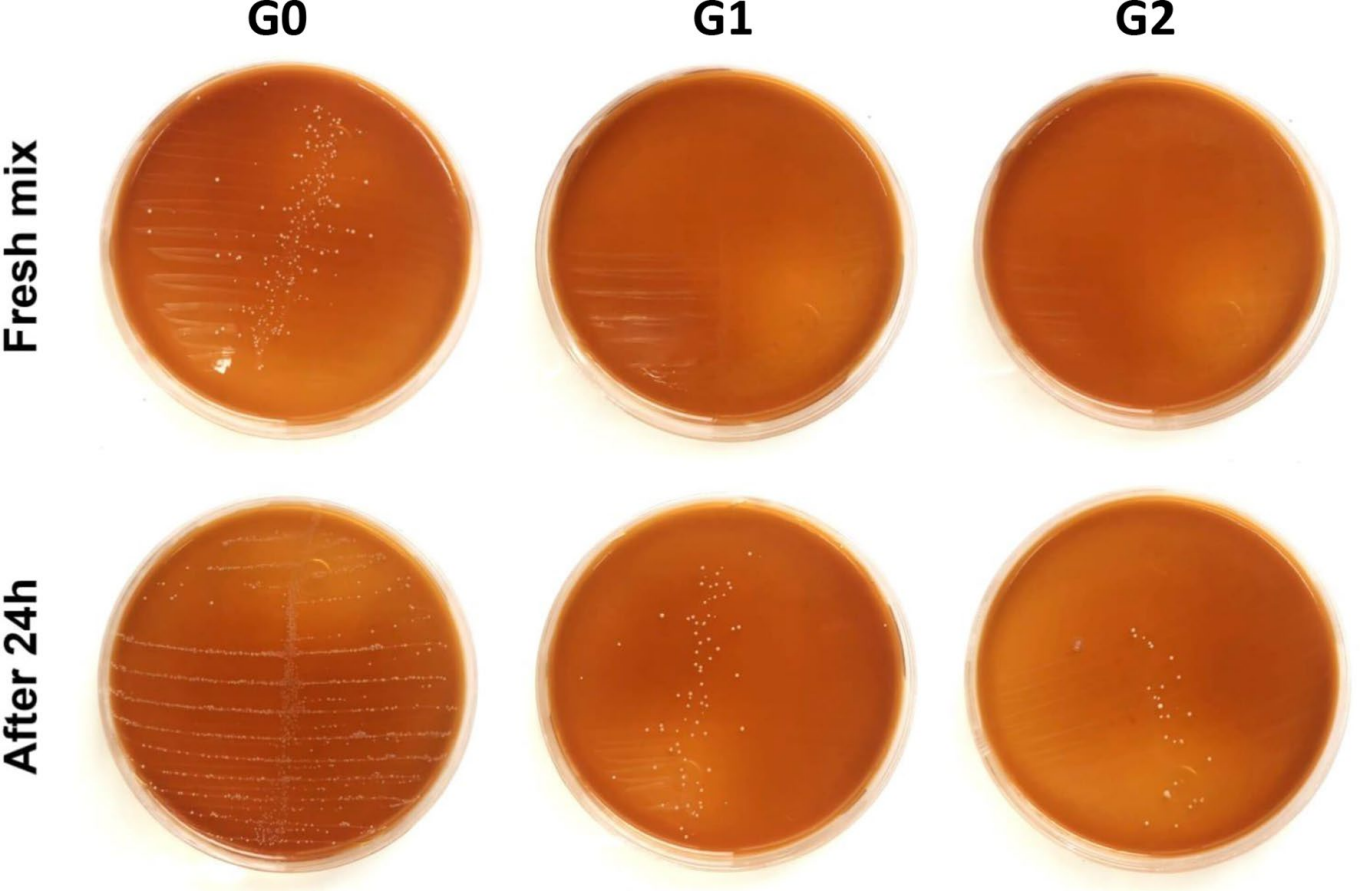
Typical photograph shows that G0 has high number of formed *E. faecalis* colonies, G1 & G2 show significant reduction of formed *E. faecalis* colonies

For fresh mixed sealers, Group 0 (Control) showed a high number of formed bacterial colonies (198.57 ± 29.11) compared with Groups 1 & 2 which showed no growth of colonies (0.00 ± 0.0). After 24 h, Group 0 still had the highest number of colonies (1142.86 ± 198.81), though increased compared to the fresh mix. Group 1 showed a limited number of colonies (32.29 ± 2.69), and Group 2 even less (16.07 ± 3.49) with insignificant difference between Group 1 and Group 2. Moreover, increasing the percentage of included fillers in RCS resulted in a statistically insignificant decrease in number of colonies as shown in Fig. 4.

**Table 1:** Means, standard deviations and results of One-Way ANOVA and post hoc test of *E- faecalis* colonies (CFU/ml) on RCS groups

	Group 0 RCS + 0wt% fillers (control group)	Group 1 RCS + 1 wt% fillers	Group 2 RCS + 2 wt% fillers	*P* value One Way ANOVA
	Mean ± SD			
Fresh mix	198.57 ^a^± 29.11	0.00 ^b^± 0.00	0.00 ^b^± 0.00	0.0001*
After 24 h	1142.86 ^a^ ± 198.81	32.29 ^b^± 2.69	16.07 ^b^± 3.49	0.0001*
P value Paired t test	0.0001*	0.0001*	0.0001*	

*Significant difference as *P* < 0.05

Means with different superscript letters per row were significantly different as *P* ≤ 0.05

### Mineralization ability

**Fig. 5:**
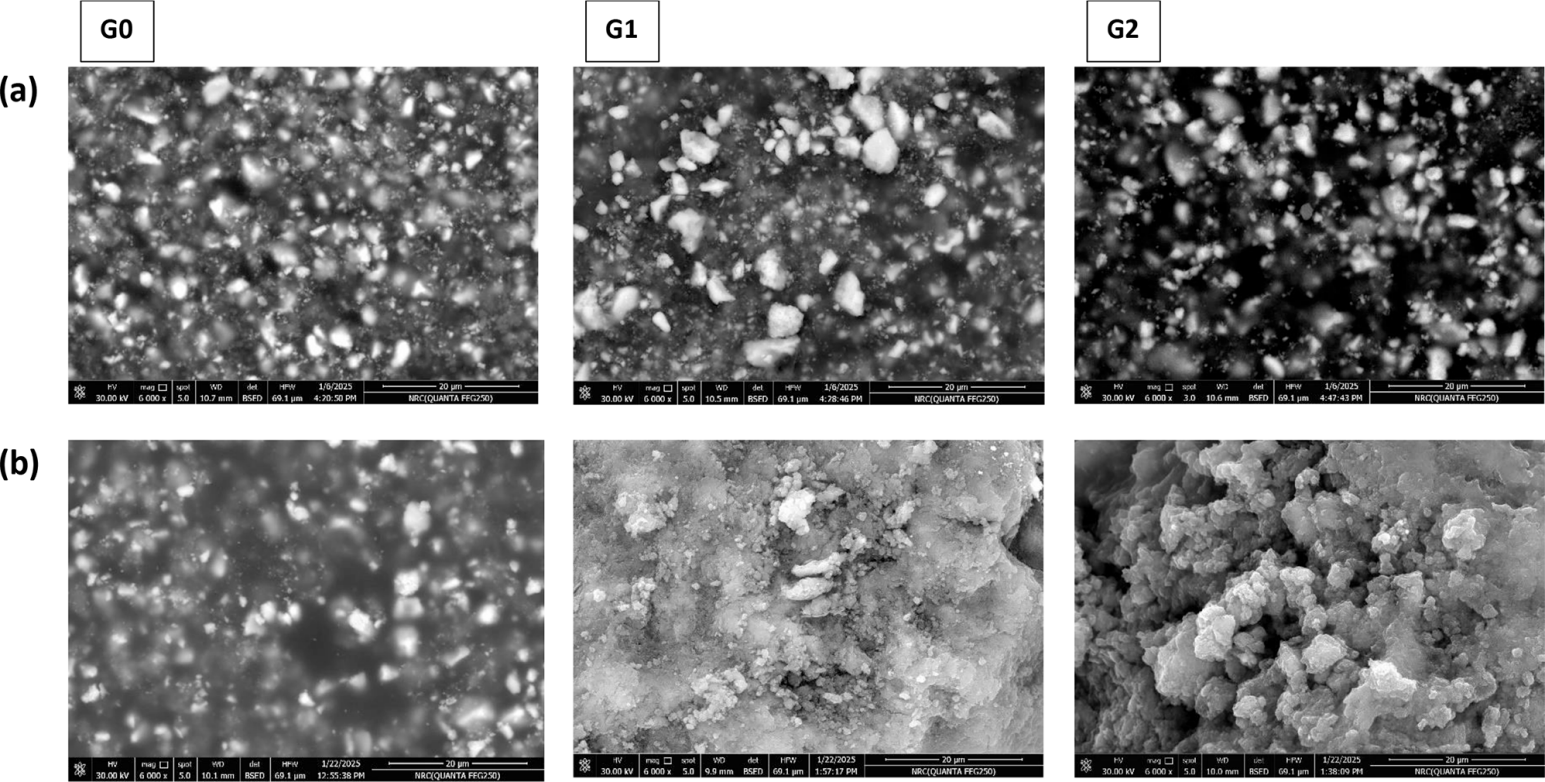
Scanning electron microscopy (SEM) images of surface of sealer specimens after immersion in SBF (**a**) immersion for 14 days (**b**) immersion for 28 days

**Fig. 6:**
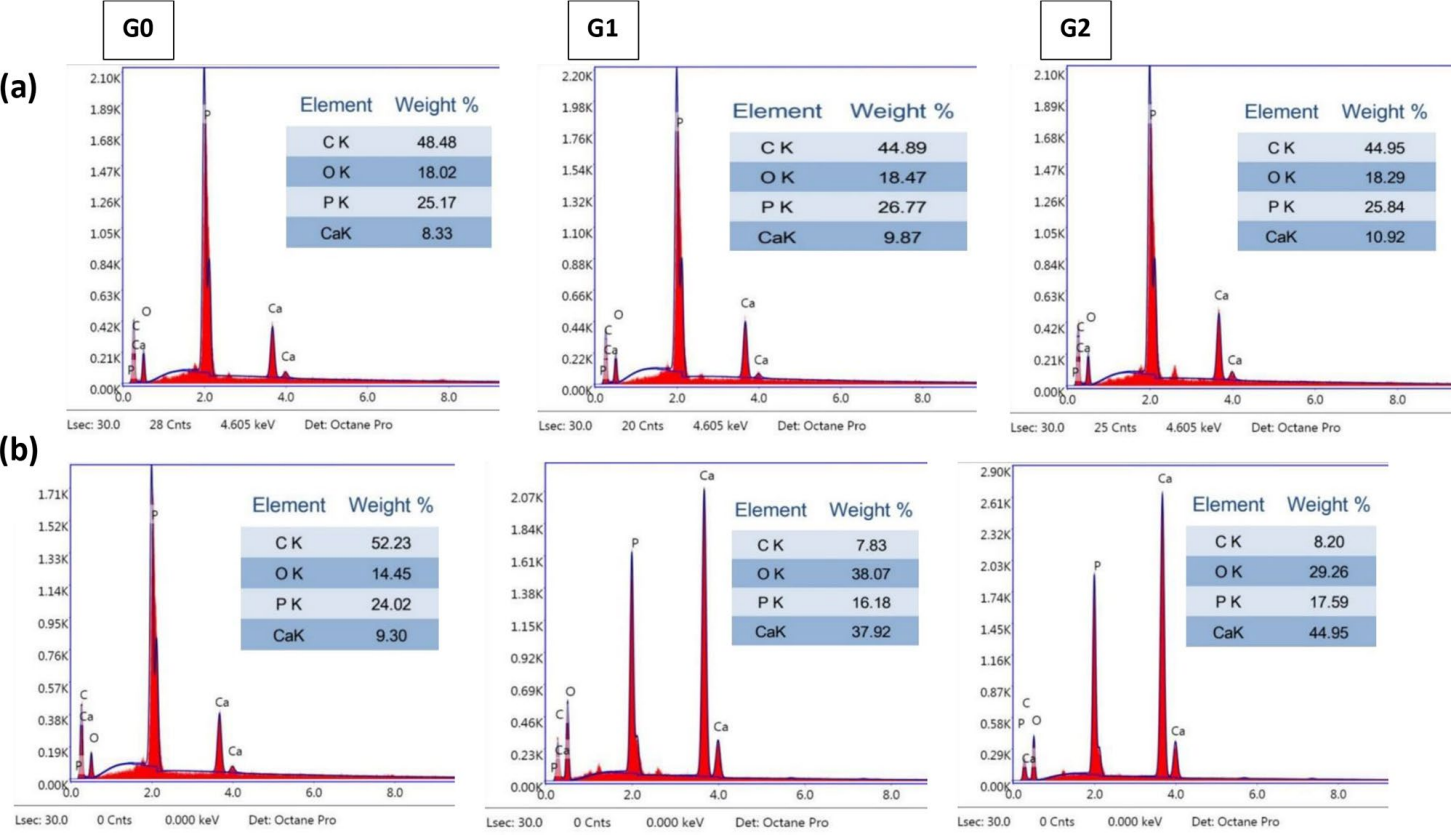
Energy-dispersive X-ray spectroscopy (EDX) mapping analysis of surface of sealer specimens after immersion in SBF (**a**) immersion for 14 days (**b**) immersion for 28 days

Regarding the results of the bioactivity test, after 14 days of immersion in SBF, SEM images showed that HA crystals formation was not clear on the surface of the modified RCS groups (G1 and G2) but the surface element analysis using EDX showed very little increase in calcium element compared to control group (G0). In contrast, after 28 days, the modified RCS groups’ surfaces (G1 and G2) had an overspreading dense layer of HA, which was confirmed by EDX, as it showed a marked increase in calcium element while the control group’s surface (G0) showed no change (Fig. 5, 6).

### Wettability evaluation

**Fig. 7:**
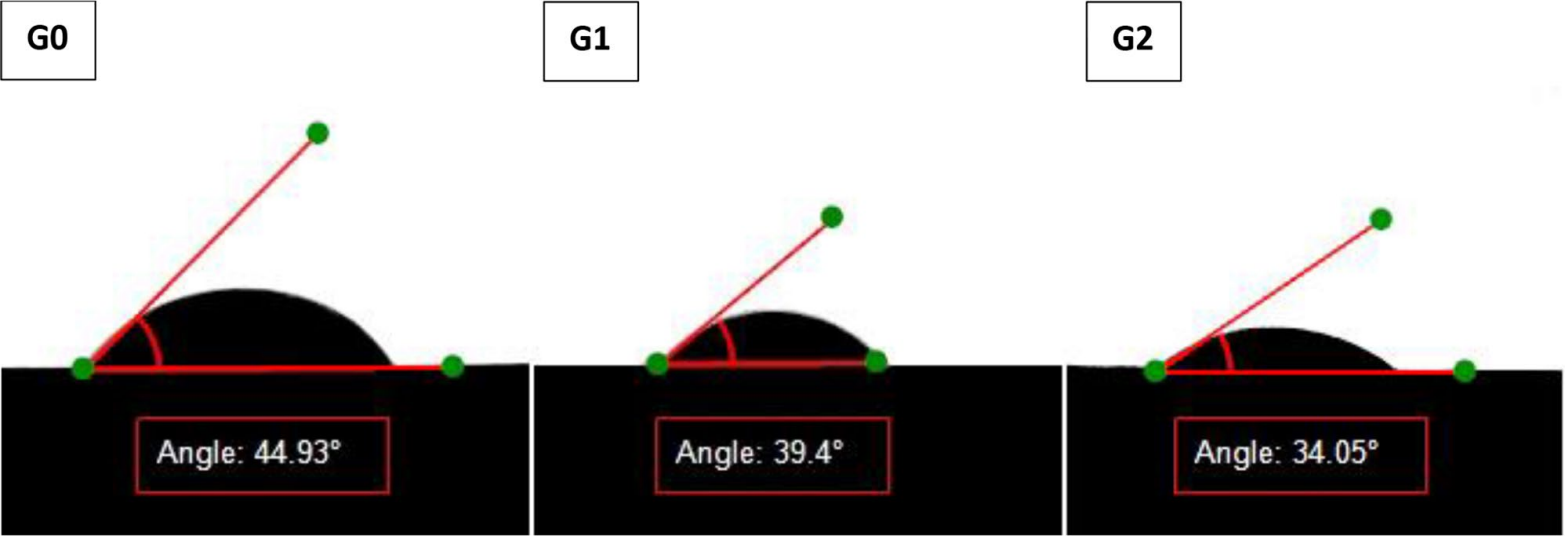
Photograph of contact angles of different sealers’ groups on root dentin surface measured by sessile drop technique

Regarding the wettability of unmodified RCS (G0) and modified RCS (G1 and G2), the means and standard deviations of the contact angle are shown in Table 2. The contact angle measurement test proved that the inclusion of HA-PDA-Ag-PDA fillers in RCS (G1 and G2) provided a statistically significant increase of RCS wettability to dentin surface (*p* < 0.05). Group 0, exhibited the highest mean contact angle (45.10° ± 0.61), indicating the lowest wetting ability among the groups. In contrast, Group 1 demonstrated a reduced mean contact angle of (39.14° ± 0.24), Group 2 showed significant decrease in contact angle of (33.75° ± 1.15), indicating the highest wetting ability (Fig. 7).

**Table 2:** Means, standard deviations and results of One-Way ANOVA and post hoc test of RCS groups’ contact angle (degree°)

	Minimum	Maximum	Mean ± SD	*P* value
Group 0	44.37	46.19	45.10 ^a^ ± 0.61	0.0001*
Group 1	38.85	39.52	39.14 ^b^ ± 0.24	
Group 2	31.30	34.88	33.75 ^c^ ± 1.15	

*Significant difference as *P* < 0.05

Means with different superscript letters per column were significantly different as *P* ≤ 0.05

## Discussion

In endodontic therapy, reliable microbiological eradication can be accomplished by mechanical instrumentation and irrigation usually in accessible areas. However, endodontic microorganisms and biofilms frequently remain in inaccessible regions, such as accessory canals, isthmi, and dentinal tubules despite cleaning, shaping, and the use of potent antimicrobial treatments [31]. Furthermore, the root canal biofilms characteristics make bacterial eradication more challenging than in the case of planktonic bacteria [31, 32]. Thus induced development of a novel RCS exhibiting antibacterial capabilities that flow into inaccessible areas and assist in the eradication of the residual endodontic microbes, resulting in greater endodontic therapy success rates. The ability of the RCS to reinforce and remineralize the structures of tooth root is another extremely desirable property. Utilizing different irrigant solutions, including NaOCl, CHX, and EDTA, which aid in dissolution of organic tissues, elimination of germs, and removal of debris, may result in dentin demineralization and decrease dentin hardness, which may increase the risk of root fracture [10, 33]. In addition, tiny gaps left inside the canal system might lead to endodontic failure, filling these gaps by deposition of remineralizing ions could improve the sealing capacity [13]. 

In previous studies, resin-based RCSs, because of their outstanding physicochemical characteristics and biological reactivity, were modified by incorporation of various nanoparticles to improve their antibacterial and remineralizing capabilities [34, 35]. Hence, AgNPs have been shown to have antibacterial activity against various kinds of microbes [36]. Furthermore, remineralization potential of nano-HA has been proven with previous studies [37, 38]. A RCS that contains triple bioactive agents of AgNP, nano-HA, and PDA was evaluated in this work. The innovation of this work lies in the combination of these agents in four layered composite, then incorporating it in the root canal sealer. This combination enhances the effect of each component where PDA could encourage the development of biomimetic hydroxyapatite. In addition, PDA can be used for in situ synthesis of Ag nanoparticles and its strong adhesion ability provide a straightforward and eco-friendly method to in situ bind Ag nanoparticles with diverse substrates throughout mild reaction which is an effective way to prevent Ag nanoparticles aggregation enhancing their stability and antibacterial activity [39]. However, there has been no report on the effect of these combined triple agents on the antibacterial, remineralization and wettability properties of RCS. Firstly, nano-hydroxyapatite was synthesized with the wet chemical precipitation method as it is a widely utilized approach owing to its low cost and capacity to synthesize a significant amount of HAp [40]. XRD confirmed successful synthesis of crystalline HA powder as the positions of diffraction peaks correspond exactly to the PDF card no. 09-0432 of HAp.[28]Then, a PDA coating is formed on the nano-HA particles through self-oxidative polymerization process which is simple and can be done at room temperature. PDA in situ reduced silver nitrate to produce silver nanoparticles using catechol and amine groups to create a coating of silver nanoparticles, via the polydopamine-assisted electroless silver metallization process, which is stabilized by another PDA coat.

The FTIR analysis verifies the formation of these multi-layered fillers. AgNPs have a large surface area, as shown in TEM images. According to the Ostwald–Freundlich equation, the dimensions and morphology of AgNPs influence the generation of silver ions. Smaller AgNPs with spherical or quasi-spherical morphology have a higher potential for the discharge of silver ions owing to their higher surface area [41]. Thus, the clustering of AgNPs restricts the discharge of silver ions, and this agglomeration can be prevented by the use of capping agents, which enhance the dissolution of AgNPs and regulate the release rate of AgNPs giving a sustainable antibacterial activity [42]. TEM image also shows the PDA coating which acts as a capping agent.

Regarding the antibacterial activity of the modified RCS, the findings of this study demonstrated that modified RCS reduce significantly E. faecalis growth in only 1 wt% concentration with no need to increase the concentration of fillers. Thus, HA-PDA-Ag-PDA fillers incorporation into the RCS markedly suppresses the proliferation of E. faecalis, a primary etiological agent of endodontic failure and persistent periodontitis. The antibacterial activity of modified RCS may be ascribed to AgNPs. One of the mechanisms that explains bactericidal action is their ability to discharge silver ions (Ag^+^). They establish compounds with nucleic acids and shatter them. Owing to the affinity and electrostatic attraction to sulfur proteins, silver ions bind to cell wall and cytoplasm, making them greatly permeable and resulting in bacterial casings disruption. The uptake of free silver ions by cells inhibits respiratory enzymes, leading to a disruption in adenosine triphosphate (ATP) release and the production of reactive oxygen species (ROS), which is an essential factor in aggravating the destruction of the cell membrane and DNA alteration. Furthermore, silver ions can effectively inhibit the production of protein through denaturing the cytoplasmic ribosomal constituents [36]. Moreover, AgNPs interfere with microbial signal transmission might significantly induce cell death and impede cellular proliferation [43]. 

The SBF immersion test is widely utilized to assess the bioactivity of biomaterials [44]. The capacity to form apatite is considered a significant indicator of RCS bioactivity. After 28 days of ageing specimens in SBF, SEM images revealed HA precipitation on the surfaces of the experimental groups in varying degrees and the underneath sealer was no longer detectable. However, the control group showed no HA precipitation. The SEM findings were verified using EDX elemental analysis of the coating’s chemical composition, yielding results that corresponded with the SEM observations. The EDX examination indicated a marked increase in calcium element on the surface of the experimental groups with Ca/P ratio higher than the stoichiometric Ca/P ratio of hydroxyapatite (Ca/*P* = 1.67). Higher Ca/P ratio suggests calcium deposition on the surface, which may result in the required bioactivity.

The bioactivity of the experimental RCS may be attributed to PDA and nano-hydroxyapatite particles. Nano-HA particles act as a crystal nuclei to enhance crystal formation and growth by continuous attraction of considerable amounts of Ca and P ions from the surrounding medium leading to biomimetic mineralization of the adjacent tissues as the composition and configuration of the synthetic apatite are identical to those of biological hydroxyapatite [45, 46]. Additionally, they serve as a supply of Ca and P, resulting in a local supersaturation state for tooth remineralization [47]. However, HA nucleation and development cannot be initiated by collagen matrix, according to a previous study, which revealed little remineralization [48]. PDA can effectively promote dentin remineralization. The mechanism of this process is that PDA surfaces have numerous catecholamine groups that have a dual function. These groups have the capacity to adhere firmly to the substrate’s surface. However, catecholamine groups that do not stick to the substrate can react with Ca^2+^ to increase the surface concentration of Ca^2+^ and aid in the production of mineralized crystals [49]. This phenomenon is called polydopamine-assisted hydroxyapatite formation. So, PDA can stimulates dentin remineralization by binding to collagen fibrils and presenting new nucleation sites enhancing biomimetic remineralization [50]. 

Furthermore, the degree of substrate surface hydrophilicity controlled mineral nucleation and growth, because greater hydrophobicity of substrates increases the interfacial energy between them and the formed nuclei. Nucleation and growth proceeded faster on the hydrophilic surface. Thus, PDA’s super-hydrophilicity makes it a suitable substrate for mineral nucleation [51]. The attainment of a fluid-tight seal relies on the wetting properties of RCS to function effectively as binding agents between the dentinal walls and the core obturating material. To achieve strong adhesion, the sealer must make intimate contact with the substrate. This promotes molecular attraction and facilitates more penetration for chemical or micromechanical surface interlocking [52]. The findings of this study showed that HA-PDA-Ag-PDA fillers inclusion into the RCS significantly increases the wetting ability of RCS to dentin surface. This may be attributed to super-hydrophilicity of PDA. Its extremely high hydrophilicity is related to the amine and hydroxyl-rich groups. The incorporation of PDA into a matrix strengthens its hydrophilic trait [53, 54]. Thus, the null hypothesis is rejected where the incorporation of HA-PDA-Ag-PDA filler increased significantly the antibacterial, mineralization and wettability properties of the resin-based RCS.

Although the current study was limited to in-vitro testing, its promising results would encourage the authors to proceed with the in-vivo phase. Additional research is required to examine biofilm growth on modified root canal sealers. Moreover, further investigations are needed to evaluate the modified RCS’s antimicrobial action against other resistant root canal pathogens. Furthermore, more research is required to assess the influence of the released calcium and phosphate ions from the sealer on the remineralization of root dentin and its ability to withstand fractures. The flow property of the modified sealer should be further studied to evaluate its ability to penetrate complex anatomy of the root canal.

## Conclusions and recommendations

In the present study, silver-polydompamine modified hydroxyapatite fillers were prepared successfully using a simple, mild and environmentally friendly approach. The prepared fillers were blended into a resin-based endodontic sealer, hoping to develop a biologically active material. The current findings showed that HA-PDA-Ag-PDA fillers could enhance the antibacterial effect of RCS against E. faecalis bacteria. In addition, the developed RCS could exhibit a remineralization potential and improved wettability to the dentin surface, creating a more stable tooth-material interface.

Based on the results of the current study, authors would highly recommend performing further in-vivo studies, in the near future, to evaluate the long-term clinical effectiveness of the prepared experimental root canal sealer.

## Data Availability

The datasets used and/or analyzed during the current study are available from the corresponding author on reasonable request.

## Abbreviations

RCS: Root canal seale
HA-PDA-Ag-PDA: Silver polydopamine-modified hydroxyapatite
FTIR: Fourier transformation infrared
XRD: X-ray diffraction
TEM: Transmission electron microscope
E. faecalis: Enterococcus faecalis
ERBSs: Epoxy resin-based Sealer
AgNPs: Silver nanoparticles
EDTA: Ethylenediamine tetra acetic acid
PDA: Polydopamine
Mefps: Mussel-adhesive-foot-proteins
Nano-HA: Nanohydroxyapatite
BHI: Brain Heart Infusion
MDCT: Modified direct contact test
CFU: Colony forming units
SBF: Simulated body fluid
SEM: Scanning electron microscope
EDX: Energy dispersive X-ray Spectroscopy
